# Improved Activation toward Primary Colorectal Cancer Cells by Antigen-Specific Targeting Autologous Cytokine-Induced Killer Cells

**DOI:** 10.1155/2012/238924

**Published:** 2012-03-19

**Authors:** Claudia Schlimper, Andreas A. Hombach, Hinrich Abken, Ingo G. H. Schmidt-Wolf

**Affiliations:** ^1^Department of Neurosurgery, University of Bonn, 53127 Bonn, Germany; ^2^Center for Molecular Medicine Cologne and Department I of Internal Medicine, University of Cologne, 53901 Cologne, Germany; ^3^Center for Integrated Oncology Cologne-Bonn, Germany; ^4^Department of Internal Medicine III, University Hospital Bonn, Sigmund-Freud-Straße 25, 53105 Bonn, Germany

## Abstract

Adoptive therapy of malignant diseases with cytokine-induced killer (CIK) cells showed promise in a number of trials; the activation of CIK cells from cancer patients towards their autologous cancer cells still needs to be improved. Here, we generated CIK cells *ex vivo* from blood lymphocytes of colorectal cancer patients and engineered those cells with a chimeric antigen receptor (CAR) with an antibody-defined specificity for carcinoembryonic antigen (CEA). CIK cells thereby gained a new specificity as defined by the CAR and showed increase in activation towards CEA^+^ colon carcinoma cells, but less in presence of CEA^−^ cells, indicated by increased secretion of proinflammatory cytokines. Redirected CIK activation was superior by CAR-mediated CD28-CD3*ζ* than CD3*ζ* signaling only. CAR-engineered CIK cells from colon carcinoma patients showed improved activation against their autologous, primary carcinoma cells from biopsies resulting in more efficient tumour cell lysis. We assume that adoptive therapy with CAR-modified CIK cells shows improved selectivity in targeting autologous tumour lesions.

## 1. Introduction

Although a variety of therapeutic options for metastatic colon cancer were evaluated during the last decade, most patients in advanced stages of the disease have no hope for cure by standard therapies. Alternative therapeutic approaches including immunotherapy are currently explored [1]. One of the major pitfalls in the adoptive immunotherapy of cancer is the strikingly low activation of T cells from cancer patients compared to healthy donors due to reduced expression of TCR/CD3 components [2]. The need for alternative effector cells in targeting colorectal carcinoma becomes obvious by the fact that T cells infiltrating colon cancer metastases have reduced CD3*ζ* chain expression and lack tumour-specific activation [3]. Compared to firstly activated effector T cells, *ex vivo* generated cytokine-induced killer (CIK) cells have a number of advantages since they exhibit properties different from effector or central memory T cells, that is, CIK cells are activated in an MHC-independent fashion [4, 5], produce proinflammatory cytokines, mainly IFN-*γ* and IL-4 [6, 7], and exhibit antigen-independent cytolytic activities against a variety of tumour cells. CIK cells are generated *ex vivo* by extensive stimulation of CD3^+^ CD56^−^ CD8^+^ T cells with IFN-*γ* and CD3 and prolonged propagation in presence of high-dose IL-2 [4]. After 2-3 weeks in culture, the majority of cells exhibit a large granular lymphocyte morphology and express both NK and T-cell markers including CD8, CD11a, CD49d, CD56, and NKG2D, while lacking most NK-cell-associated activating and inhibitory receptors [8]. The CD45RA^+^ CCR7^−^ CD62L^(+)^, CD27^+^, CD28^−^, MIF-1a^+^ CIK phenotype coincides with that for terminally differentiated memory T cells [9]. CIK cells display extraordinary cytolytic capacities toward a broad array of malignant cells [10] and traffic efficiently to the tumour side after systemic delivery [11]. Upon activation, CIK cells upregulate perforin and FasL as well as DAP10 which couples NKG2D signaling to perforin-based cytotoxicity [12], thereby recognizing a class of stress-associated ligands, NKG2D ligands, expressed on the tumour cell surface. Consequently, CIK cells exhibit MHC-unrestricted cytotoxicity and do not rely on a particular antigen. Based on these and other properties, CIK cells attracted interest for adoptive immunotherapy particularly in advanced stages of the disease where repression of MHC expression or defects in the antigen-processing machinery frequently occur. For application in adoptive therapy, CIK cells display the advantage that they do not require priming but can rapidly be expanded in culture [13] and are less associated with graft-versus-host disease than conventional effector T cells [14]. CIK cells have been adoptively transferred in phase I trials to treat leukemia/lymphoma and various solid tumours including hepatocellular carcinoma, colon carcinoma, astrocytoma, melanoma, and renal cell carcinoma [15–17]. CIK therapy showed low toxicity [18], however, limited therapeutic efficacy; CIK therapy is consequently assumed to require large numbers of CIK cells to be transferred to achieve efficient tumour clearance.

In this situation, we asked to improve CIK cell activation against autologous tumour cells. We therefore made use of the concept to redirect T cells towards defined target cells by a recombinant chimeric antigen receptor (CAR) which is expressed on the surface of T cells and provides both antigen-targeting specificity and T-cell activation [19]. The CAR in the extracellular moiety is composed of a single-chain fragment of variable region (scFv) antibody for target binding and in the intracellular moiety of the CD3*ζ* signaling chain to initiate T-cell activation upon binding. To furthermore increase T-cell activation, the costimulatory CD28 endodomain was linked to CD3*ζ* in a combined signaling moiety [20]. We here demonstrate that *ex vivo* generated CIK cells from colon carcinoma patients can be engineered with a tumour-specific CAR; such “designer” CIK cells increase cytokine secretion and cytolysis when engaging autologous, primary colon carcinoma cells. Data suggest such CAR-engineered CIK cells to improve the antitumour response in the adoptive immunotherapy of colon carcinoma.

## 2. Materials and Methods

### 2.1. Patient Characteristics and Evaluation

Patients with colorectal carcinoma were treated by surgery of the primary tumour lesion. Approval of the local ethics committee was obtained. Diagnosis of CEA^+^ colorectal carcinoma was confirmed by immunohistology in a pathology reference centre.

### 2.2. Cells, Cell Lines, and Reagents

T cells were isolated from heparinized peripheral blood by Ficoll density centrifugation. CIK cells were generated as previously described [21]. In brief, nonadherent peripheral blood mononuclear cells were stimulated in RPMI 1640 medium, 10% (v/v) FCS, and 25 mM HEPES with human recombinant IFN-*γ* (1,000 U/mL; Roche Biochemicals, Mannheim, Germany). After 24 h, 50 ng/mL OKT3 monoclonal antibody (mAb) (Orthoclone; Cilag, Sulzbach, Germany), 100 U/mL IL-1*β*, and 300 U/mL IL-2 (Roche, Mannheim, Germany) were added. Cells were propagated in a density of 3 × 10^6^ cells/mL in presence of IL-2. Primary colon carcinoma cell cultures were established from patients' carcinoma specimens obtained during surgery as described [22]. In brief, tissue specimens were incubated in HBSS buffer containing 100 U/mL DNase I (Roche Biochemicals), 50 U/mL collagenase III (Biochrom, Berlin, Germany), 150 U/mL hyaluronidase (Sigma, Deisenhofen, Germany), and 0,08 U/mL insulin (Hoechst, Bad Soden a. Ts., Germany) at 37°C for 15 min. Cells in the supernatant were centrifuged for 5 min at 400 × g and erythrocytes eliminated by incubation in 10 mL “erythrocyte-lysis buffer” (8,29 g/L NH_4_Cl, 1 g/L KHCO_3_, 0,0371 g/L EDTA) and for 15 min. Cells were washed and resuspended in Leibovitz medium, 10% (v/v) FCS, 1 mM L-glutamine, 1x MEM vitamins, 2.5 mg/mL transferrin, 1 g/L sodium bicarbonate, 1 g/L glucose, 80 U/mL insulin, and 10 mg/mL gentamycin (all from Gibco Invitrogen, Karlsruhe, Germany). Cultures contaminated with fibroblasts were removed. Carcinoma cells grown in culture were monitored for CEA expression by immunohistochemical analysis using an anti-CEA mAb 1C3 (Abcam, Cambridge, MA) and a peroxidase-conjugated Fab anti-mouse Ab (1 : 50) (Roche Diagnostics) and visualized by 3-Amino-9-ethylcarbazole (AEC; Sigma). 293T cells are human embryonic kidney cells that express the SV40 large T antigen. LS174T is a CEA^+^ colorectal carcinoma line (ATCC, CL-188), and Colo201 is a CEA^−^ adenocarcinoma line (ATCC CCL 224). OKT3 (ATCC CRL 8001) is a hybridoma cell line that produces the anti-CD3 mAb OKT3. 293T cells were propagated in DMEM medium supplemented with 10% (v/v) FCS, and all other cell lines were cultured in RPMI 1640 medium, 10% (v/v) FCS (all Life Technologies, Paisly, UK). OKT3 monoclonal antibody (mAb) was affinity purified from hybridoma supernatants utilizing goat anti-mouse IgG2a antibodies (Southern Biotechnology, Birmingham, AL, USA) that were immobilized on N-hydroxy-succinimid-ester-(NHS)-activated sepharose (Amersham Biosciences, Freiburg, Germany). Human IgG1 antibodies and the phycoerythrin-(PE-)conjugated anti-CD3 mAb UCHT1 were purchased from Dako, Hamburg, Germany, and the goat antihuman IgG antibody and its FITC- and PE-conjugated F(ab′)_2_ derivatives from Southern Biotechnology. The antihuman IFN-*γ* mAb NIB42 and the biotinylated anti-human IFN-*γ* mAb 4S.B3 were purchased from BD Bioscience, San Diego, CA, USA.

### 2.3. Engineering of CIK Cells and Receptor-Mediated Activation

The generation of the expression cassettes for the CEA-specific CARs BW431/26-scFv-Fc-*ζ* (no. 439) and BW431/26-scFv-Fc-CD28-*ζ* (no. 607) was previously described [20]. CIK cells were engineered with the CAR by retroviral gene transfer, and CAR expression was identified by flow cytometry utilizing a PE- or FITC-conjugated F(ab′)2 anti-human IgG1 antibody (1 *μ*g/mL), which recognizes the CAR extracellular IgG1 Fc spacer, and the anti-CD3 mAb (UCHT-1, 1 : 20). Flow cytometry was performed using an FACScan cytofluorometer equipped with the CellQuest research software (Becton Dickinson, Mountain View, CA, USA). Engineered CIK cells were cocultivated in round bottom 96-well microtiter plates (1.25−10 × 10^4^ engineered cells/well) with CEA^+^ and CEA^−^ tumour cells (5 × 10^4^ cells/well), respectively. After 48 hrs, culture supernatants were analyzed by ELISA for IFN-*γ*. Briefly, IFN-*γ* was bound to the solid phase anti-human IFN-*γ* mAb NIB42 (1 *μ*g/mL) and detected by the biotinylated anti-human IFN-*γ* mAb 4S.B3 (0.5 *μ*g/mL) (both from Pharmingen). The reaction product was visualized by a peroxidase-streptavidin conjugate (1 : 10,000) and ABTS (both from Roche Diagnostics).

### 2.4. Cytotoxicity Assay

Specific cytotoxicity of receptor-grafted T cells against target cells was monitored by an XTT-based colorimetric assay. Briefly, XTT (2,3-bis(2-methoxy-4-nitro-5sulphonyl)-5[(phenyl-amino)carbonyl]-2H-tetrazolium hydroxide) reagent (1 mg/mL) (“Cell Proliferation Kit II,” Roche Diagnostics) was added to the cells and incubated for 30–90 min at 37°C. Reduction of XTT to formazan by viable tumour cells was monitored colorimetrically at an adsorbance wavelength of 450 nm and a reference wavelength of 650 nm. Maximal reduction of XTT was determined as the mean of wells containing tumour cells only, and the background as the mean of wells containing culture medium only. The nonspecific formation of formazan due to the presence of effector cells was determined from triplicate wells containing effector cells in the same number as in the corresponding experimental wells. The cytotoxicity towards tumour cells was calculated as follows: cytotoxicity [%] = 100 − {[OD (exp. wells-corresponding number of effector cells)/OD (tumour cells without effectors − medium)] × 100}. 

### 2.5. ELISpot Assay

IFN-*γ*-producing cells were determined using the human “IFN-*γ* ELISpot kit” (Hölzel, Cologne, Germany). Peripheral blood lymphocytes (4 × 10^4^ cells) were plated on nitrocellulose 96-well plates (Millipore, Bedford, MA) coated with anti-IFN-*γ* antibody. Cells were stimulated either with phytohemagglutinin (10 *μ*g/mL, Sigma) or with 100 Gy irradiated tumour cells (1 × 10^3^ tumour cells per 4 × 10^4^ CIK cells) for 48 hrs. Bound IFN-*γ* was detected by a biotinylated antibody and visualized by streptavidin alkaline phosphatase and BCIP/NBT (BioRad, Munich, Germany) as substrate. Spots were recorded using the Bioreader 2000 (Bio Sys, Karben, Germany).

### 2.6. Statistical Analyses

 Statistical analyses were performed using the two-tailed Student's *t*-test if not otherwise described.

## 3. Results

**Figure 1:**
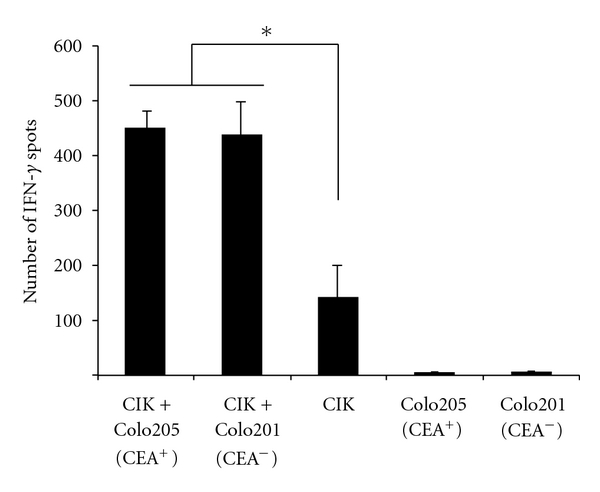
CIK cells are equally activated upon binding to CEA^+^ and CEA^−^ tumour cells. CIK cells (4 × 10^4^ cells) generated from blood lymphocytes of healthy donors were coincubated with CEA^+^ Colo205 or CEA^−^ Colo201 tumour cells (10^3^ tumour cells) for 48 hrs. IFN-*γ* production was monitored by ELIspot analysis. Data represent the mean ± standard error of the mean; a representative experiment out of three is shown. *P < 0.05.

CD3^+^ CD56^+^ CIK cells were generated *in vitro* from peripheral blood lymphocytes of a healthy donor by incubation with IFN-*γ*, IL-1beta, and the agonistic anti-CD3 mAb OKT3 and propagated in the presence of IL-2 as previously described [21]. After 2-3 weeks, CIK cells showed their characteristic repertoire of surface molecules (Table 1). CIK cells are activated upon coincubation with CEA^+^ Colo205 and CEA^−^ Colo201 colon carcinoma cells, respectively, indicated by the increase in the number of IFN-*γ*-secreting CIK cells (Figure 1). Activation of CIK cells occurred equally upon binding to CEA^+^ and CEA^−^ tumour cells confirming the known property of CIK cells of antigen-independent antitumour activation.

**Figure 2:**
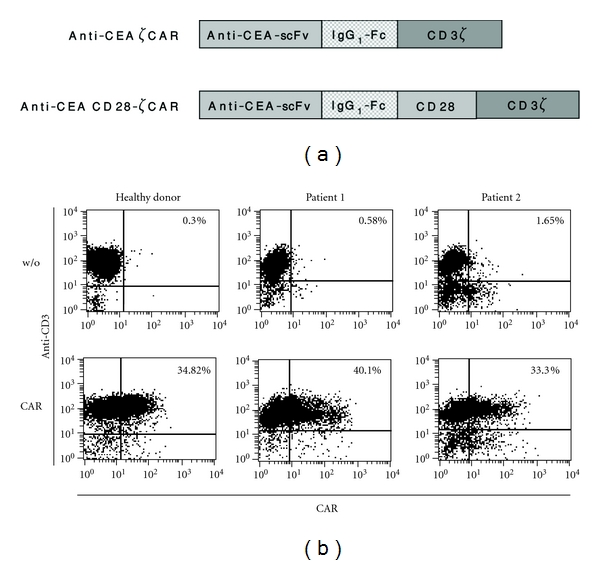
Genetic engineering of CIK cells with CARs. (a) Schematic diagram of the expression cassettes coding for the CEA-specific CAR BW431/26scFv-Fc-CD3*ζ* and BW431/26scFv-Fc-CD28-CD3*ζ* used in this study. (b) CIK cells were generated from mononuclear cells and subsequently transduced by retroviral infection to express the anti-CEA CAR. Mock-transduced cells (w/o) served as controls. CAR expression was recorded by flow cytometry using the anti-CD3 antibody OKT3 and the anti-human IgG-Fc antibody which detects the CAR extracellular IgG1 Fc spacer domain.

We asked to furthermore improve CIK cell activation selectively towards CEA^+^ colorectal carcinoma cells by engineering with a CEA-specific CAR. Therefore, CIK cells were retrovirally transduced to express either the anti-CEA CAR BW431/26scFv-Fc-CD3*ζ* with the CD3*ζ* or alternatively the anti-CEA CAR BW431/26scFv-Fc-CD28-CD3*ζ* with the combined CD28-CD3*ζ* signaling domain, both harboring the same CEA binding domain (Figure 2(a)). Both CARs were efficiently expressed on the CIK cell surface as recorded by flow cytometry using an antibody directed towards the CAR extracellular IgG1 Fc spacer domain (Figure 2(b)). CAR expression on engineered cells was similar on CIK cells from healthy donors and from colorectal cancer patients.

**Figure 3:**
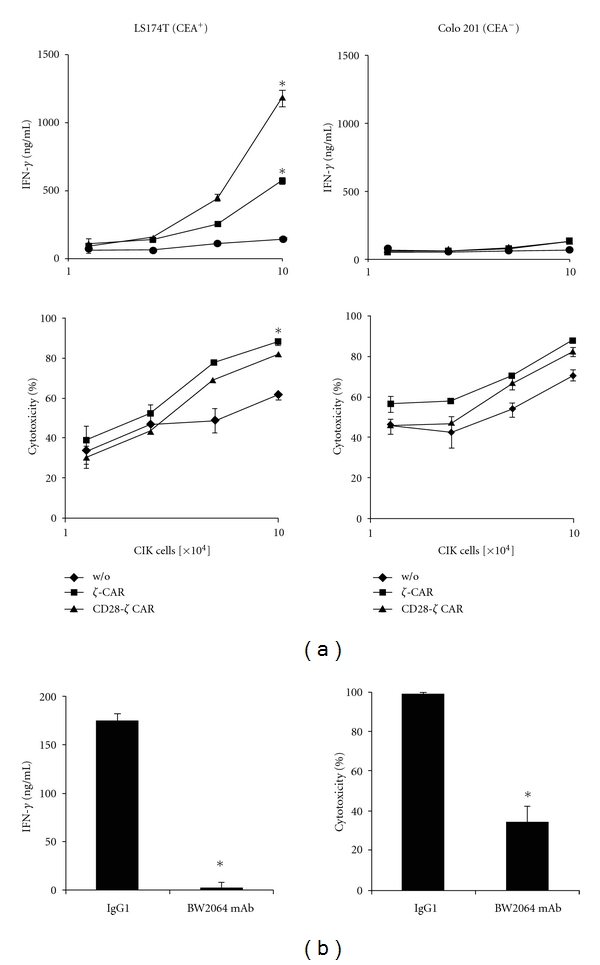
CAR engagement produces higher activation of engineered CIK cells toward colorectal carcinoma cells. (a) CIK cells were engineered with the CEA-specific *ζ* or CD28-*ζ* CAR (1–10 × 10^4^ CAR^+^ cells/well) and incubated for 48 hrs with CEA^+^ LS174T and CEA^−^ Colo201 cells (5 × 10^4^ cells/well). Mock-modified CIK cells without CAR (w/o) served as control. IFN-*γ* secreted by activated CIK cells into the culture supernatants was recorded by ELISA (upper), and cytolysis of tumour cells was monitored using the XTT-based viability assay (lower). *P < 0.05 compared to nonmodified CIK cells (w/o). (b) To block the CEA-specific CAR binding domain, CIK cells with *ζ* CAR were incubated in presence of the anti-idiotypic antibody BW2064, which binds to the CAR BW431/26-scFv domain, or with an isotype-matched IgG1 control antibody of irrelevant specificity together with CEA^+^ LS174T tumour cells for 48 hrs. IFN-*γ* secreted into the culture supernatant and specific cytotoxicity toward LS174T cells were monitored. Data show a representative experiment out of three. *P < 0.05.

In order to record antigen-redirected activation, CIK cells with engineered anti-CEA CAR were coincubated with CEA^+^ colon carcinoma cells and with CEA^−^ carcinoma cells as controls. CAR CIK cells increased IFN-*γ* secretion upon coincubation with CEA^+^, but not upon co-incubation with CEA^−^ tumour cells, whereas CIK cells without CAR did not increase IFN-*γ* secretion (Figure 3(a)). IFN-*γ* secretion was more increased when CIK cells were activated by the CD28-CD3*ζ* than the CD3*ζ* CAR. Moreover, redirected cytolytic activity of CAR-engineered CIK cells towards CEA^+^ tumour cells is increased compared to CIK cells without CAR. The cytolytic activity towards CEA^−^ tumour cells was not substantially altered by engineering with a CEA-specific CAR as control. We conclude that CIK cells can specifically be improved in activation by CAR engagement.

To confirm antigen specificity in CAR-mediated CIK activation, we blocked the CAR by incubation with the anti-idiotypic antibody BW2064, which is directed toward the BW431/26-scFv domain for antigen binding. As summarized in Figure 3(b), both IFN-*γ* secretion and cytolysis of CAR CIK cells were repressed in presence of the anti-idiotypic mAb, whereas an isotype-matched IgG1 antibody of irrelevant specificity had no effect. Data demonstrate that the antitumour activation of CIK cells is mediated by the engineered CAR in a CEA-dependent fashion.

**Figure 4:**
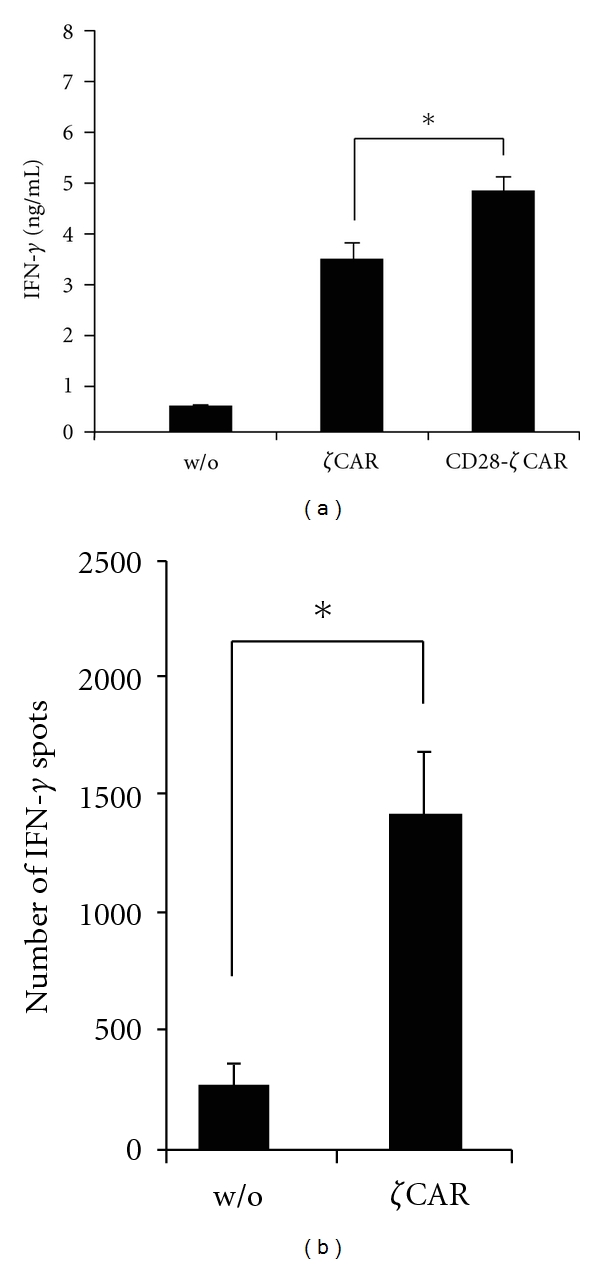
Activation of CIK cells from cancer patients against autologous colon carcinoma cells. Primary colon carcinoma cells were isolated from a colon carcinoma biopsy as described in Section 2 and cultured *in vitro* for short term. CIK cells from the same patient were generated *in vitro* and engineered with the *ζ* and the CD28-*ζ* CAR. CIK cells were coincubated for 48 hrs with the autologous CEA^+^ colon carcinoma cells (4 × 10^4^ CIK cells; 10^3^ tumour cells). Secreted IFN-*γ* in the culture supernatant was monitored by ELISA. Data represent the means of triplicates ± standard error of the mean. One representative experiment out of three is shown. *P < 0.05.

We now explored whether activation of CIK cells from colorectal cancer patients toward their autologous tumour cells can be improved by CAR-mediated engagement of target cells. Colorectal carcinoma cells were isolated from surgical specimens and confirmed by immunostainings to express CEA (data not shown). Colorectal carcinoma cells were coincubated with engineered autologous CIK cells with the CD3*ζ* or the combined CD28-CD3*ζ* CAR. As summarized in Figure 4, activation of CAR CIK cells is substantially increased against autologous tumour cells compared to nonmodified CIK cells of the same patient. Increase in IFN-*γ* secretion was higher upon stimulation by the CD28-CD3*ζ* than by the CD3*ζ* signaling CAR. Increased IFN-*γ* secretion is due to increased numbers of activated CIK cells as indicated by the increased numbers of IFN-*γ*-ELISpots. Activation is antigen specific since the same CAR-engineered CIK cells did not increase IFN-*γ* secretion in presence of CEA^−^ Colo201 cells (data not shown). Taken together activation of CIK cells from colorectal cancer patients toward autologous tumour cells can be improved by CAR engagement of target cells and is furthermore increased by combined CD28-CD3*ζ* CAR signaling.

## 4. Discussion

To improve CIK cell activation towards autologous tumour cells, we here revealed that (i) CIK cells generated *ex vivo* from peripheral blood lymphocytes can be engineered with a CAR as a targeting and activating receptor, (ii) engineered CIK cells increase activation upon CAR engagement which is superior upon CD28-CD3*ζ* signaling, and (iii) redirected by the CAR, CIK cells from tumour patients exhibit improved activation towards autologous tumour cells. While CIK cells recognize tumour cells in an antigen-independent fashion, CAR-engineered CIK cells gain antigen specificity as defined by the CAR binding domain. CAR-engineered CIK cells show improved tumour specificity indicated by increased IFN-*γ* secretion upon contact to CEA^+^ compared to CEA^−^ tumour cells, while nonmodified CIK cells do not increase IFN-*γ* in presence of CEA^+^ compared to CEA^−^ tumour cells. Improved CIK cell activation requires antigen engagement since activation is blocked by an anti-idiotypic antibody directed against the CAR binding domain. While CIK cells are susceptible to CD3*ζ* signaling, furthermore increase in IFN-*γ* secretion by designer CIK cells upon CD28-*ζ* CAR stimulation likely contributes to improve antitumour activity *in vivo* through activation of bystander cells. Cytolytic activity is predominantly mediated by perforin triggered by NKG2D in CIK cells [12]; other mechanisms may additionally contribute since blocking of NKG2D did not completely eliminate the cytotoxic activity of CIK cells [23].

To evaluate the specific situation in cancer patients, we confronted CIK cells from a colon cancer patient with the autologous colon cancer cells from a biopsy *in vitro*. Similarly as CIK cells from a healthy donor, CAR-engineered CIK cells from cancer patients showed improved activation against autologous tumour cells indicated by increase in IFN-*γ* secretion compared to CIK cells without CAR. Previous reports by Sheen et al. [24] and our group [25] demonstrated efficient targeting of CD3^+^ effector T cells from cancer patients toward autologous colon carcinoma cells; CIK cells herewith expand the panel of effector cells suitable to target autologous tumour cells.

Soluble CEA in the serum of cancer patients, particularly in advanced stages of the disease, may prevent CAR-mediated activation of engineered CIK cells by blocking the binding domain. In the case of the BW431/26 scFv CAR domain, we previously demonstrated that CEA in concentrations up to 20 *μ*g/mL does not block CAR-mediated T-cell activation and does not inhibit induction of cytolytic activities [26]. We therefore do not expect that serum CEA interferes with the activity of anti-CEA CAR-modified CIK cells in colorectal cancer patients.

Given the insufficiencies in activating CIK cells and the difficulties in generating sufficient quantities for clinical applications, improved CIK activation upon CAR signaling is assumed to decrease the numbers of CIK cells required to elicit a therapeutic response. Previous strategies to overcome limitations in specific T-cell activation used bispecific antibodies which target CD3 on effector cells and the tumour-associated antigen CA125, Her2/neu, or other tumour-associated antigens on tumour cells [27, 28]. Redirecting CIK cells from patients with ovarian cancer with bispecific antibodies increased lysis of primary ovarian cancer cells [27]. Most recently, CIK cells were modified with a CD33-specific CAR for targeting acute myeloid leukemia cells [29] and with a CD19-specific CAR with 4-1BB costimulatory signal for targeting B-lineage acute lymphoblastic leukemia [29, 30].

## 5. Conclusion

CAR-mediated redirection of CIK cells from colon carcinoma patients improves their activation towards autologous tumour cells in an antigen-dependent fashion.

## Figures and Tables

**Table 1:** Characterization of CIK cells.

Marker	Positive cells (%)
CD3	98.6
CD4	22.1
CD8	67.8
CD14	0.0
CD33	9.3
CD56	31.7
HLA-DR	67.4

CIK cells were generated from peripheral blood lymphocytes from a healthy donor as described in Section 2. After 2-3 weeks of propagation, cells express the phenotype of CD3^+^CD56^+^ CIK cells. Data of a representative CIK cell induction are shown.
